# Interleukin-10 Enhances the Intestinal Epithelial Barrier in the Presence of Corticosteroids through p38 MAPK Activity in Caco-2 Monolayers: A Possible Mechanism for Steroid Responsiveness in Ulcerative Colitis

**DOI:** 10.1371/journal.pone.0130921

**Published:** 2015-06-19

**Authors:** Violeta Lorén, Eduard Cabré, Isabel Ojanguren, Eugeni Domènech, Elisabet Pedrosa, Arce García-Jaraquemada, Miriam Mañosa, Josep Manyé

**Affiliations:** 1 Inflammatory Bowel Disease Unit, Research Institute of Health Sciences ‘Germans Trias i Pujol’, Badalona, Barcelona, Spain; 2 CIBER, Madrid, Spain; 3 Inflammatory Bowel Disease & G-I Unit, Department of Gastroenterology, ‘Germans Trias i Pujol’ University Hospital, Badalona, Barcelona, Spain; 4 Department of Pathology, ‘Germans Trias i Pujol’ University Hospital, Badalona Barcelona, Spain; Institute of Pathology, GERMANY

## Abstract

Glucocorticosteroids are the first line therapy for moderate-severe flare-ups of ulcerative colitis. Despite that, up to 60% of patients do not respond adequately to steroid treatment. Previously, we reported that low IL-10 mRNA levels in intestine are associated with a poor response to glucocorticoids in active Crohn’s disease. Here, we test whether IL-10 can favour the response to glucocorticoids by improving the TNFα-induced intestinal barrier damage (assessed by transepithelial electrical resistance) in Caco-2 monolayers, and their possible implications on glucocorticoid responsiveness in active ulcerative colitis. We show that the association of IL-10 and glucocorticoids improves the integrity of TNFα-treated Caco-2 cells and that p38 MAPK plays a key role. *In vitro*, IL-10 facilitates the nuclear translocation of p38 MAPK-phosphorylated thereby modulating glucocorticoids-receptor-α, IL-10-receptor-α and desmoglein-2 expression. In glucocorticoids-refractory patients, p38 MAPK phosphorylation and membrane desmoglein-2 expression are reduced in colonic epithelial cells. These results suggest that p38 MAPK-mediated synergism between IL-10 and glucocorticoids improves desmosome straightness contributing to the recovery of intestinal epithelium and reducing luminal antigens contact with *lamina propria* in ulcerative colitis. This study highlights the link between the intestinal epithelium in glucocorticoids-response in ulcerative colitis.

## Introduction

Ulcerative colitis (UC) and Crohn’s disease (CD), collectively known as Inflammatory Bowel Disease (IBD), are chronic inflammatory illnesses mainly occurring in Western societies, but with increasing incidence in developing countries. As they are chronic diseases with no definitive cure, they have a great impact on patients’ quality of life and account for a great consumption of health resources.

Glucocorticoids (GC) are very often used for the treatment of active CD and still remain the first line therapy for moderate-severe attacks of UC [1]. In spite of that, up to 60% of patients do not adequately respond to steroid treatment [2]. Lack of response does not diminish the side effects of GC and implies the use of more aggressive and expensive rescue therapies.

Previous studies have suggested that defects in intestinal *lamina propria* T cell apoptosis are related to the pathogenesis of CD [3]. Studies by our group have shown that CD patients who did not respond to GC have a decreased intestinal T and B cell apoptosis, as compared to GC responders [4]. However, other mechanisms could also account for steroid resistance in IBD. Steroid treatment failure has been related to the some polymorphisms in the multidrug resistance-1 (*MDR-1)* gene [5], resulting in increased amounts of the drug detoxifying P-glycoprotein 170 (Pgp-170), which decreases the intracellular levels of GC [5]. Other studies have focused on the GC receptor (GCR) -α function, either because of impaired expression or by blockade by GCR -β [6–8].

Although most immunosuppressive actions of GC have been reported on immune cells [9,10], there are evidences proving that they also act on the intestinal epithelial cells. Enhanced epithelial uptake of glucose following oral administration of dexamethasone does not occur in GCRα-deficient mice, demonstrating the involvement of epithelial cells in GC-induced actions [11].

Recently, our group has reported that low IL-10 mRNA levels in intestinal baseline biopsies are associated with a poor response to GC in active CD [4]. Furthermore, deficiency of this immune regulatory cytokine in animal models is associated with increased intestinal permeability and development of spontaneous colitis [12]. In intestinal epithelial cells, IL-10 is able of reducing TNFα-induced endoplasmic reticulum stress [13]. Also, other members of the IL-10 family, such as IL-22, also have beneficial effects on intestinal epithelial cells through the activation of STAT-3 [14].

As a whole, all these data suggest that part of the anti-inflammatory effects of IL-10 could be accounted for their actions on the intestinal epithelium, by regulating the barrier function. However, the molecular mechanisms of these actions, and how they affect GC response, are not well understood. Therefore, in the present study we wanted to test whether the presence of IL-10 can favour the response to GC by improving the TNF-α-induced intestinal barrier damage in confluent Caco-2 cell monolayers. In addition, the possible implications of some of the *in vitro* findings on GC responsiveness in UC were investigated in a series of biopsy samples from GC sensitive and refractory UC patients.

## Methods

### Materials

Dulbecco's modified Eagle's medium, fetal bovine serum, nonessential aminoacids, penicillin, streptomycin, rhTNFα, rhIL-10, LDS Sample Buffer (4x), 10x reducing agent, 4–12% SDS-PAGE gels, Alexa Fluor 555 Goat anti-mouse, Alexa Fluor 488 Donkey anti-mouse, Hoechst 33342, and TaqMan Assays (Life Technologies, Paisley, UK); p38 MAP Kinase inhibitor (SB203580) (InvivoGen, San Diego, CA, USA); Tris-HCl, NaCl, NP-40 detergent, deoxycholic acid sodium salt (DOC), sodium dodecyl sulfate (SDS), Na_3_VO_4_, Dimetil Sulfóxido (DMSO), phenylmethyldulfonyl fluoride, NaF, Protease Inhibitor Cocktail (P8340), Trichloroacetic acid, Tris-base, glycine, methanol, DPX mounting media, paraformaldehyde, sucrose, Bovine Serum Albumin (BSA), glutaraldehyde, NaOH, Sodium cacodylate, HCl, osmium tetroxide, toluidine blue, uranil acetate, lead citrate and Mouse Anti-α-tubulin (clone B5-1-2) (Sigma-Aldrich, St. Louis, MO, USA); Blocking buffer Odyssey, Donkey anti-mouse IRDye800CW and Donkey anti-rabbit IRDye680 (LI-COR, Lincoln, NE, USA); Mouse Anti-phospho-p38 MAPK (Thr180/Tyr182) (28B10), Rabbit Anti-p38 MAPK (Cell Signaling Technology, Beverly, MA, USA); Mouse Anti- Desmoglein (DSG)-2 (Acris antibodies, Herford, Germany); miRNeasy Mini Kit (Qiagen, Gaithersburg,MD, USA); PrimeScript RT reagent (Perfect Real Time) kit (TAKARA, Otsu, Japan); fluoroprep mounting media (Biomerieux, Marcy d’Etoile, France); tEpon 812 embedding kit (Tousimis, Rockville, MD, USA); Methyl-prednisolone (as a GC) (Urbason, Sanofi, Paris, France). Caco-2 cell line (ECACC, UK) was kindly provided by Dr. A. Río (Research Institute of Health Sciences ‘*Germans Trias i Pujol*’ of Badalona, Spain).

### Epithelial cell culture

The human colon cell line Caco-2 was used for *in vitro* assays. This cell line was cultured in Dulbecco's modified Eagle's medium containing 10% fetal bovine serum, 2% nonessential amino acids, 100 U/ml penicillin, and 100 μg/ml streptomycin and kept in a humidified incubator at 37°C with 5% CO_2_. Media of Caco-2 cells were changed 2 times per week. Cells were grown to confluence on 24-well cellulose transwell inserts 0.4 μm pore size, high density (BD Falcon, Franklin Lakes, NJ, USA) and 21 days after seeding, Caco-2 cells spontaneously differentiate into a highly functionalized epithelial barrier exhibiting similar structural and biochemical characteristics as mature enterocytes [15][16][17]. Then TNF-α (100 ng/mL), GC (50 μM), IL-10 (20 ng/ml) and/or SB203580 (20 μM in DMSO) were added to the basal medium, depending on the experimental conditions, during 48h with one medium change at 24h. SB203580 inhibits only the activity but not the phosphorylation of p38 MAPK, by binding to its catalytic residues (ATP binding domain) [18]. The resulting groups were: TNF, TNF_GC, TNF_IL10, TNF_GC_IL10, TNF_GC_IL10_SB203580, and a control group (medium alone) was also included. Each experimental condition was replicated five times.

### Intestinal biopsies collection

The colonic samples used in this study were collected from a single-center, prospective, observational study on Cytomegalovirus (CMV) infection in UC patients [19]. Demographic and clinical data were recorded. CMV-free UC patients were divided into two groups according to their response to GC: a) GC-responsive group (n = 8 subjects) consisted of active UC patients admitted for the treatment of an acute flare requiring iv steroid treatment (1 mg/kg/day of prednisolone or equivalent); b) GC non-responsive group (n = 7 subjects) consisted of active UC patients refractory to steroid treatment. GC-refractoriness was defined as the absence of clinical improvement after a course of 7 to 10 days of 1 mg/kg/day of iv prednisolone, in which case iv Cyclosporine A (4 mg/kg/day) was started.

Patients were included immediately prior to or during the first 24 hours after starting steroids or Cyclosporine A. Patients with UC limited to the rectum, those with previous episodes of colonic CMV disease, neoplasia, immunodeficiency diseases, and systemic steroid treatment 30 days before the inclusion were excluded from the study.

Rectal biopsies were obtained by colonoscopy or flexible sigmoidoscopy with gentle insufflation and immediately routinely processed for paraffin embedding. These samples were stored properly in the *Germans Trias i Pujol* BioBank (Badalona, Spain) to guarantee their conservation. In active UC patients, tissue samples were taken from the centre of the largest mucosal ulcers, when possible. The sample collection in UC groups was performed at the same time of patient inclusion (pre-treatment) and also after 7–10 days of steroid treatment (post- treatment).

The study protocol was previously approved by the ‘Germans Trias i Pujol' hospital Ethics Committee (06/03/1999, Badalona). Patients were included after obtaining their written informed consent.

### Assessment of Intestinal Epithelial Barrier Function

Transepithelial electrical resistance (TEER) was used as a measure of paracellular permeability and barrier function in confluent Caco-2 cell monolayers. To evaluate the stimulus effect on the epithelial barrier, TEER was measured at baseline and after 48-h of stimulation, by means of a Millicell-ERS epithelial volt-ohm meter (Millipore, Billerica, MA, USA). The blank measurements (transwell without cell monolayer) were subtracted from TEER values of each experimental condition and also were adjusted for the filter surface (0.3 cm^2^), and the values were expressed as ohms (Ω) per cm^2^. The results represent the percentage of final TEER increases respect of their basal TEER value.

### Western blot analyses

Western blot of 48h-incubation extract from homogenized Caco-2 cell monolayers was performed in a RIPA buffer (25 mM Tris-HCl at pH 8.0, 150 mM NaCl, 1% NP-40, 1% DOC, 0,1% SDS, 1 mM Na_3_VO_4_, 1 mM phenylmethyldulfonyl fluoride, 1 mM NaF and 10 ul/ml protease inhibitor cocktail). The cellular extracts were incubated during 45 minutes with gentle shaking and centrifuged at 16,000 *g* for 15 minutes. The supernatants obtained were precipitated by 100% Trichloroacetic acid-2% DOC method and denaturized in 1x LDS sample buffer and 1x reducing agent at 70°C for 10 minutes. Subsequently, the samples were separated on 4–12% SDS-PAGE gels at 200 V, and electrotransferred at 100 V for 90 minutes onto nitrocellulose membranes in 27 mM Tris-base, 197 mM glycine and 20% methanol (pH 8.2) buffer. After, the membranes were blocked and were incubated overnight at 4°C with specific antibodies (Anti-phospho-p38 MAPK 1:250 (v/v); Anti-p38 MAPK (total p38 MAPK) 1:250 (v/v); Anti-α-tubulin 1:6000). Donkey anti-mouse IRDye800CW and Donkey anti-rabbit IRDye680 (1:20000 (v/v)) were used to detect primary antibodies. Fluorescent signal were captured by Odyssey scan (LI-COR, Lincoln, NE, USA) and each protein was quantified by O*dyssey Infrared Imaging software* (v3.0) and normalized to α-tubulin. The results are expressed as a ratio to the control group to normalize the band intensity between nitrocellulose membranes.

### Real-time PCR

Total RNA was isolated from Caco-2 cells homogenized with trizol using the *RNeasy Mini* Kit according to the manufacturer’s instructions. The RNA integrity was evaluated by Experion system (BioRad, Hercules, CA, USA) and only were used those samples with a RNA Quality Index ≥ 7. cDNA was generated using *PrimeScript RT reagent* (*Perfect Real Time* kit). In brief, 800ng of total RNA were mixed with oligo dT primer, PrimeScript RT Enzyme and Random 6 Mers, and the retrotranscription was carried out into a thermocycler (NyxTechnik ATC401; San Diego, CA, USA).

Real-time PCR was performed using TaqMan Assays (IL-10Rα_Hs00155485-m1; IL-10Rβ_Hs00175123-m1; GCRα_Hs00353740; DSG2_Hs00170071-m1; DSC2_Hs00951428-m1; E-Cadherin_Hs01023894-m1) and the results were normalized with GAPDH as housekeeping (GAPDH_Hs02758991-g1). The PCR thermal cycling was as follows: initial denaturing at 95°C / 10 seconds, 40 cycles of 95°C / 15 seconds, and 60°C / 1 minute. Thermal cycling was performed using a LightCycler 480 system (Roche Diagnostics, Basel, Switzerland). Folds changes respect to control group were calculated by 2^-ΔΔCt^.

### Immunohistochemistry

Paraffin embedded biopsy samples from UC patients were used to evaluate the phospho-p38 MAPK. Four μm histological sections were deparaffinised and rehydrated in xylene/ethanol solutions. Antigen retrieval was done by boiling in citrate buffer (pH 6) for 45 minutes and the endogen peroxidase was inhibited by hydrogen peroxide (3%) for 10 minutes. The primary antibody anti-phospho-p38 MAPK at 1:50 (v/v) was revealed with Ultraview Universal DAB kit, and counterstained with Harris's haematoxylin. Immunostaining was carried out in an automatic *BenchMark XT* station (Ventana Medical Systems, Oro Valley, AZ, USA). Slides were then dehydrated and mounted in DPX mounting media. Negative controls were obtained by omitting primary antibody and a mammary gland tissue was used as a positive control to test the antibody specificity. Between 10–20 slides fields were examined by a pathologist blind to the samples codes using a Axioskop 2 light microscope (Carl Zeiss S.A., Oberkochen, Germany) and DFC480 digital camera (Leica Microsystem, Cambridge, UK) to capture images. The relative quantification of positive nuclei (p38 MAPK^+^) was made with the following score: 0- No positive nuclear staining; 1- few labelled nuclei; 2-many labelled nuclei; 3- all or almost all labelled nuclei.

### Immunofluorescence evaluations

Caco-2 cells were fixed with fresh ice-cold 4% paraformaldehyde / 2% sucrose for 40 minutes at 4°C and following permeabilized with methanol at -20°C to localized intracellular phospho-p38 MAPK. After, the cells were blocked with 3% BSA 30 minutes at room temperature, and incubated overnight at 4°C with primary antibody (anti-phospho-p38 MAPK 1:100 (v/v) in 1% BSA / PBS buffer), or without antibody as negative control (data not shown). Alexa fluor 555 Goat anti-mouse was used as secondary antibody (1:200 (v/v) in 1%BSA/PBS as buffer) and Hoechst stain (0.4 μg/ml) was added for nucleus counterstain. Samples were mounted with fluoroprep mounting media and assessed within the next 24 hours using a fluorescence microscope with apotome module (Zeiss AxioObserver Z1) and AxioCam MRc 5 camera. At least 10 individual fields were captured randomly and analyzed with AxioVision software (v 4.8.1). The results were expressed as percentage of cells with nuclear or/and cytoplasmic staining.

DGS2 intracellular localization from paraffin-included intestinal biopsies of UC patients was assessed. Sample preparation and antigen retrieval for fluorescent staining were the same as immunochemistry protocol. Anti-DSG-2 (1/50 (v/v) 1% BSA / PBS as buffer) was added as primary antibodies. The slides were incubated 1 hour at room temperature with Alexa Fluor 488 Donkey anti-mouse (1:100 in 1%BSA/PBS as buffer) secondary antibody. Right now, the protocol was the same as the Caco-2 cells. Results were quantified using a score from 0 to 3 depending on the paracellular staining intensity.

### Transmission electron microscopy

Paracellular gaps were detected by transmission electron microscopy. In short, Caco-2 cell monolayers were fixed with Karnovsky buffer (pH 7.4) for 5 hours. After that, the cells were washed with cocodilate buffer (0.1 M, pH 7.4), and fixed with 1% osmium tetraoxide for 1h. Then the samples were dehydrated in graded alcohols, and embedded in a mixture of propylene oxide and Epon 812 resin at different concentration. Semi-thin 0.5 μm-sections were stained by toluidine blue to select the region of interest, and 0.1 μm ultra-thin sections were stained with 5% uranil acetate/citrate. The ultra-thin sections were examined and photographed using a JeoL-1011 electronic microscope (Jeol Ltd., Tokyo, Japan). The results expressed the number and intercellular gaps localization in each experimental condition.

### Statistical Analysis

Data are expressed as median with IQR or as means ± SEM. TEER, western blot, real-time PCR, immunohistochemistry and immunofluorescent results were analysed using Mann-Whitney test. The changes during the treatment course in UC patients were evaluated with Wilcoxon test. To paracellular gap evaluations by transmission electron microscopy used Chi-Square test (X^2^). All statistical analyses were performed and all graphs were generated with SPSS.15 (SPSS Inc.). Values of p≤0.05 were considered to be statistically significant.

## Results

### The association of IL-10 and GC improves the integrity of TNF-treated intestinal epithelial cells *in vitro*

To assess the effect of GC and IL-10 on the epithelial layer, we conducted experiments on Caco-2 cell monolayers by measuring the TEER as a surrogate of the integrity of the confluent cell monolayer, as well as the strength of paracellular junctions. Measures were made before and after addition of GC, IL-10 or both, either in the presence or absence of TNF-α in the culture medium.

**Fig 1:**
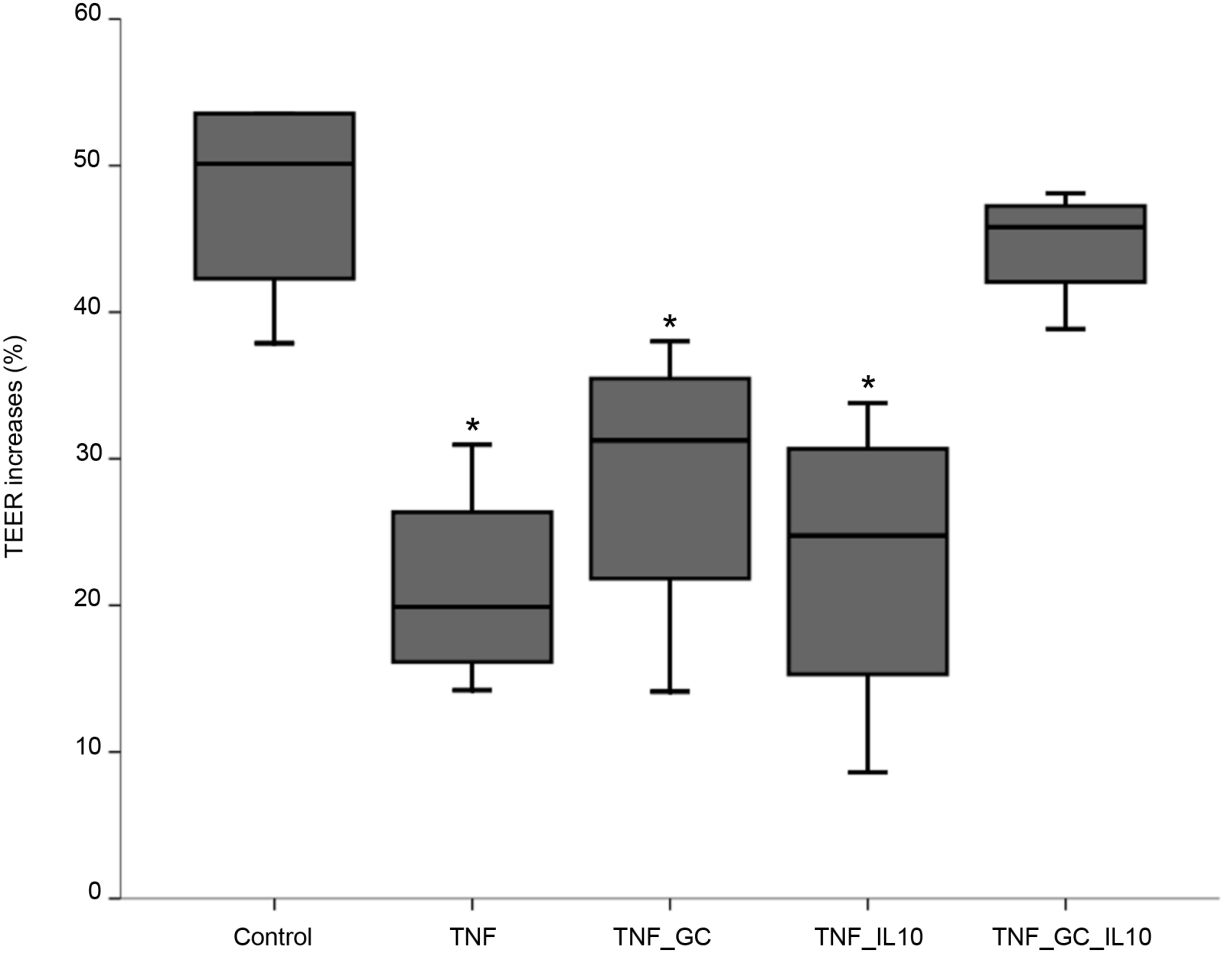
IL-10 and GC restore control TEER values in Caco-2 cell monolayers. Figure represents median values (IQR, limits) of percent TEER changes in Caco-2 cell monolayers after 48h of incubation with different stimuli. *p ≤ 0.02 *vs* control and TNF_GC_IL10.

In the absence of TNF-α, no differences in TEER value among groups were found (median (IQR): control -5.9 (-10.5, 4.5); GC -4.39 (-25.2, 3.71); IL10–6.8 (-13.2, 8.5); GC_IL10 7.9 (-20.7,14.1)). However, a significant decrease of TEER was observed in the presence of TNF-α as compared to control conditions (Fig 1). Such a decrease was not reversed either by GC or IL-10 alone, but returned to control values when both agents were simultaneously added to the medium (Fig 1).

### p38 MAPK plays a key role in the synergistic actions of IL-10 and GC on the epithelium

**Fig 2:**
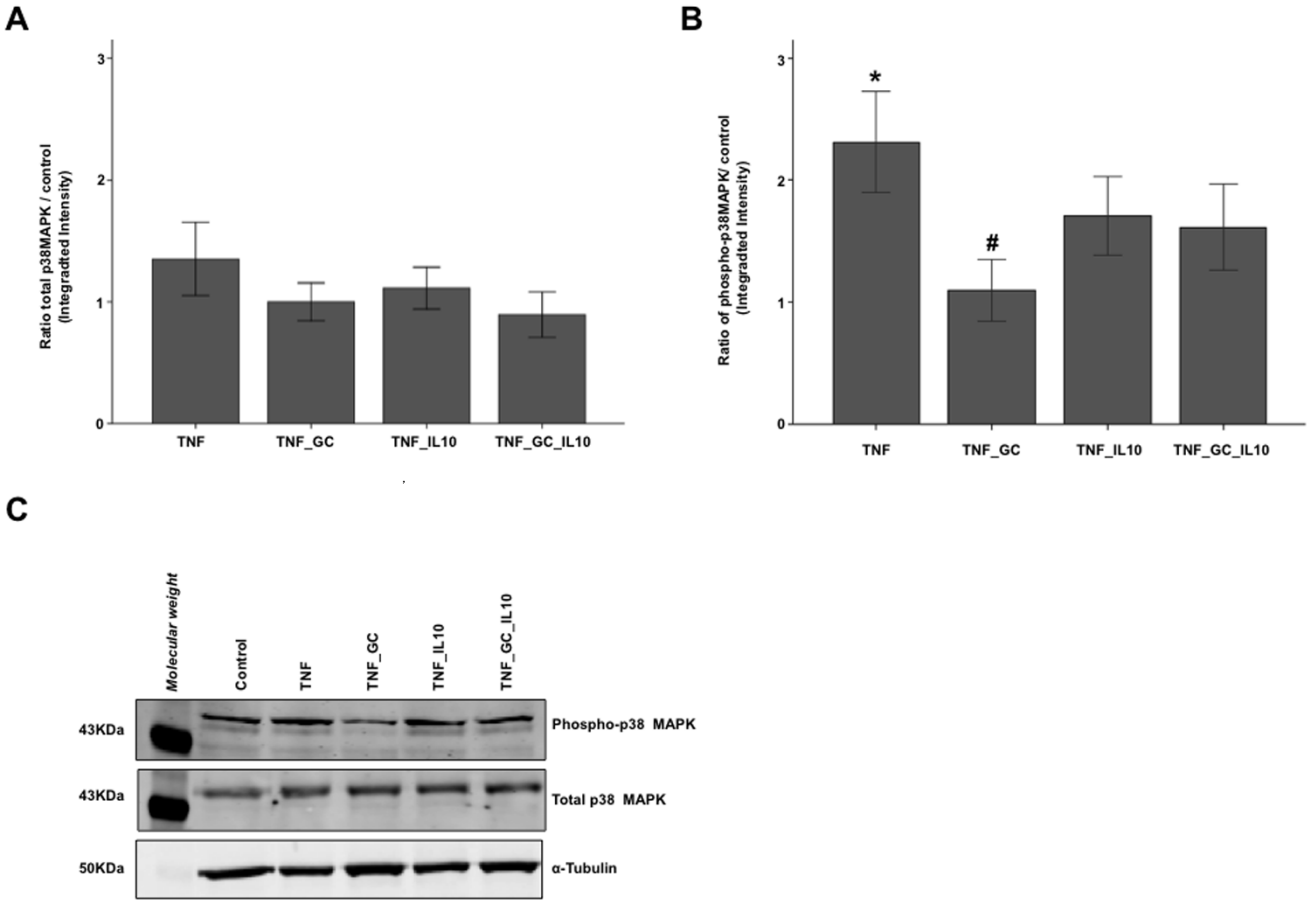
IL-10 reverts the GC-mediated decrease in phosphorylated-p38 MAPK activity. Figure shows (median (IQR, limits)) the ratio for total p38 MAPK (Panel A) and phosphorylated p38 MAPK (Panel B) versus control group in TNF-treated Caco-2 cell monolayers. An example of Western blot bands obtained for these proteins and α-tubulin (as housekeeping protein) is provided in Panel C. *p≤ 0.026 *vs*. all other groups, ^#^p = 0.028 *vs*. TNF_IL10 and TNF_GC_IL10;.

**Fig 3:**
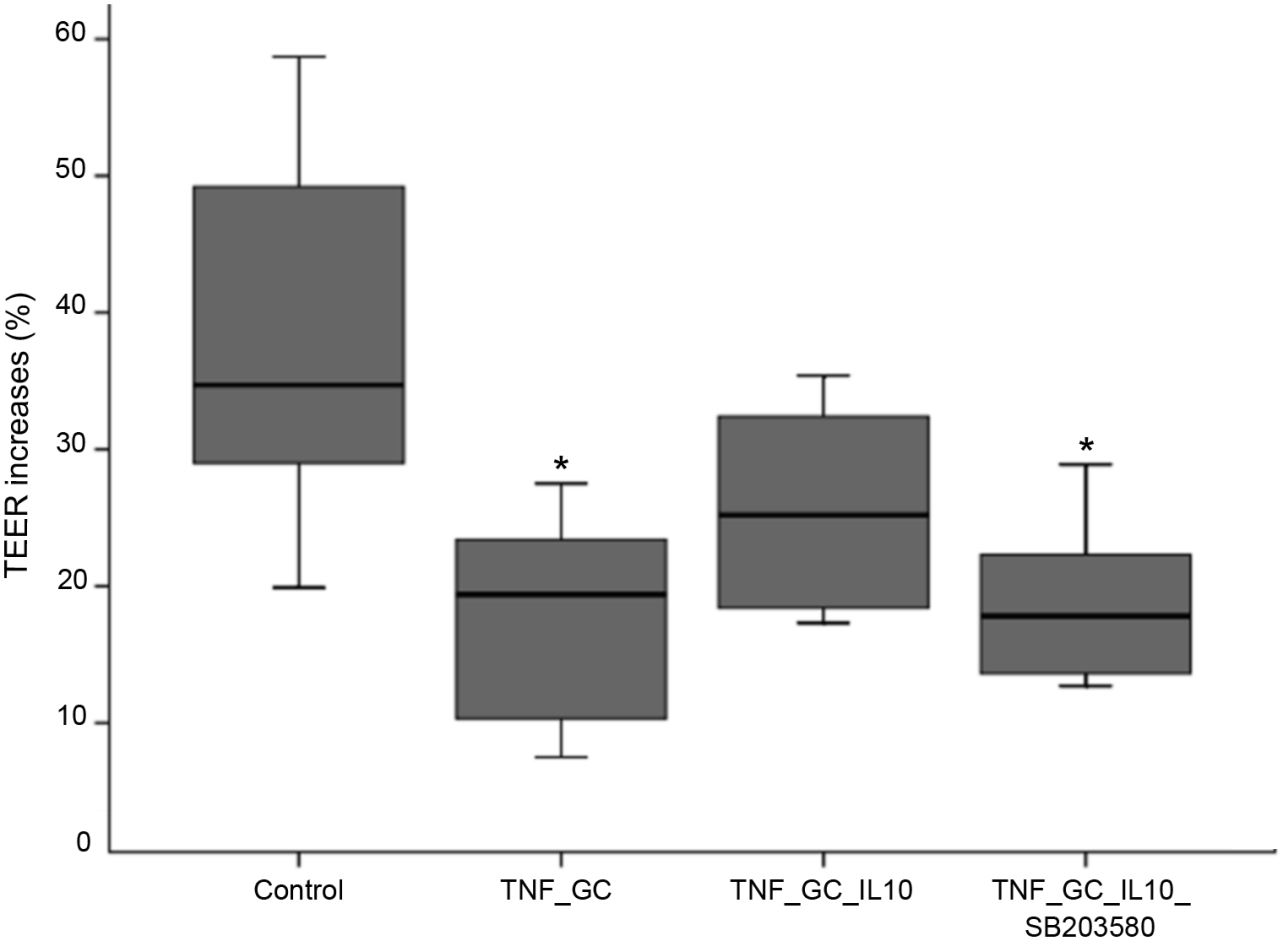
SB203580 reverses the synergistic action of IL-10 and GC on TEER in Caco-2 cell monolayers. Values are median (IQR, limits) of percent TEER changes in Caco-2 cell monolayers after 48h of incubation with different stimuli. *p≤ 0.042 *vs* control.

In order to explore the mechanisms of the above-mentioned effect of IL-10 and GC, several proteins of both intracellular signalling pathways and tight junctions (TJ) were measured by Western blot in cell lysates of the same above groups. No changes were found in the TJ proteins (*zonula occludens* (ZO)-1 and occludin), among groups (data not shown). The total amount of p38 MAPK were similar to control condition in all TNF-treated groups (Fig 2A). On the other hand, the presence of IL-10 –alone or associated to GC—resulted in intermediate levels of phospho-p38 MAPK (Fig 2B). To ascertain the role of p38 MAPK in the action of GC and IL-10 on TEER, a second series of experiments was carried out comparing TEER among controls, TNF_GC, TNF_GC_IL-10, and a fourth group with TNF, GC, IL-10 and an inhibitor of p38 MAPK activity (SB203580) (Fig 3). As in the previous experiment, the association of GC and IL-10 reversed the reduction of TEER to control values, but this effect did not occur in the presence of the inhibitor of p38 MAPK activity (Fig 3).

### IL-10 facilitates the nuclear translocation of phosphorylated p38 MAPK in the presence of TNF-α and GC

**Fig 4:**
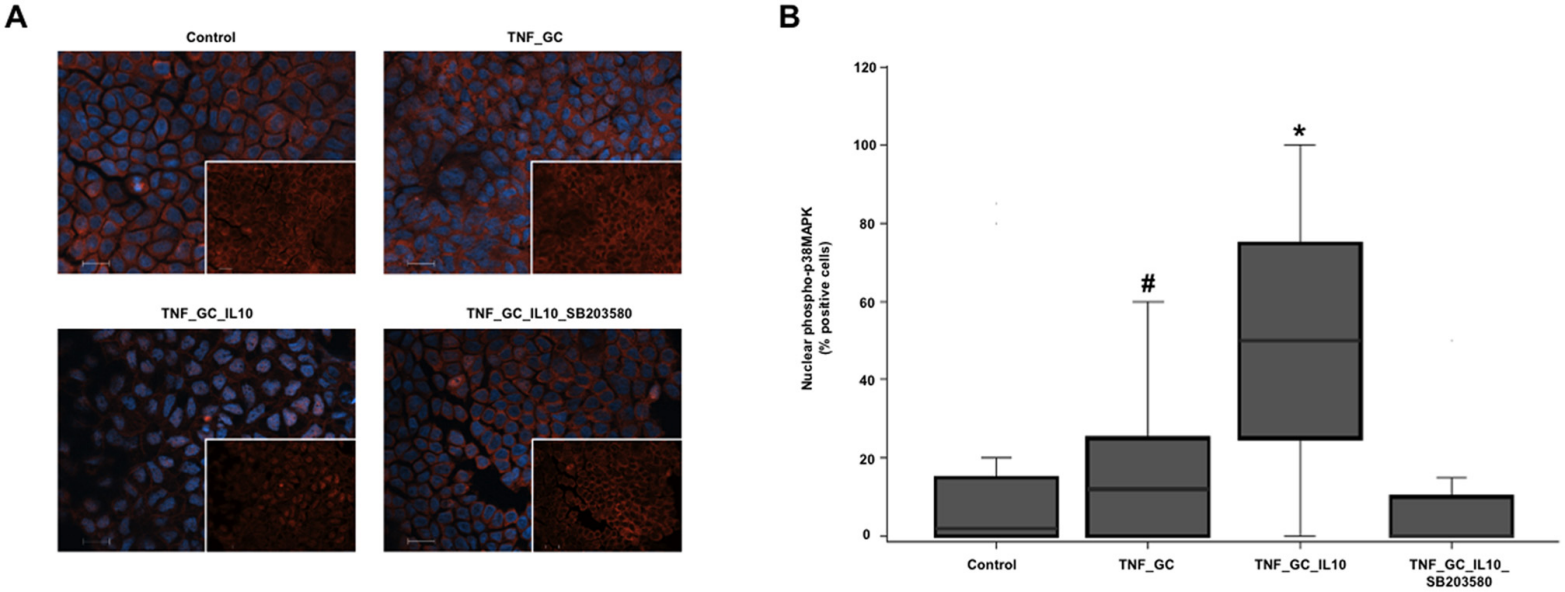
Both IL-10 and GC facilitate the phosphorylated-p38 MAPK nuclear translocation in Caco-2 cell monolayers. Panel A shows the nuclear arrangement of phosphorylated p38 MAPK in cultured Caco-2 cell monolayers, by immunofluorescence staining with anti-phospho-p38 MAPK (Thr180/Tyr182) (red) and Hoechst dye (blue). Scale bar 20 μm. White squares show images without Hoechst staining. Panel B shows the median (IQR, limits) percentage of cells with positive phospho-p38 MAPK nuclear staining. *p≤ 0.021 *vs*. other groups, #p≤ 0.043 *vs*. TNF_GC_IL-10_SB203580.

Immunofluorescence staining of Caco-2 cell monolayers allowed to verify that the presence of IL-10 in the culture medium, together with TNF-α and GC, facilitated nuclear translocation of phospho-p38 MAPK, an effect that was reversed by adding the p38 MAPK SB203580 inhibitor (Fig 4).

### IL-10-induced p38 MAPK activation modulates the expression of GCR-α, IL-10 R-α and DSG-2 in Caco-2 cell monolayers

**Fig 5:**
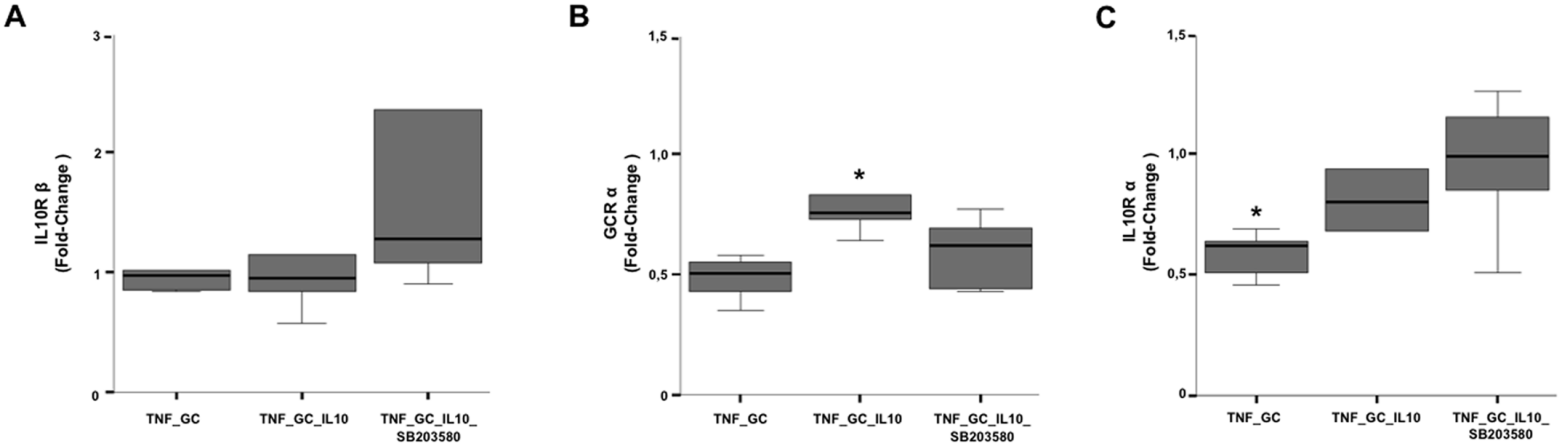
IL-10 plus GC increases GCR-α (p38 MAPK-dependent) and IL-10R-α expression in Caco-2 cell monolayers. Graphs represent fold change values (2^-AACt^) of IL-10R- β (Panel A), GCR-α (Panel B) and IL-10R-α (Panel C) expression, with respect to their basal condition (control or DMSO group). *p≤ 0.04 *vs* other groups.

With the aim to investigate the consequences of IL-10-induced p38 MAPK activation, we studied the expression of potentially implicated genes in the same *in vitro* experimental groups of confluent monolayers of Caco-2 cells. First, we measured the expression of GCR -α and -β, IL-10R-α and -β, as well as IL-10 itself. Also, the expression of E-cadherin, DSG-2, and Desmocolin-2 (DSC-2), as proteins involved in adherens junctions (AJ) and desmosomes, were evaluated (since we had failed to disclose any change in TJ-related proteins). Expression of IL-10 and GCR-β was absent in all experimental conditions, while expression of IL-10R-β—although present—showed no differences among groups (Fig 5A). However, GCR-α and IL-10R-α expression were significantly higher in the presence of IL-10 together with TNF-α and GC, as compared to the cell monolayers treated only with TNF-α and GC (Fig 5B and 5C). The addition of the p38 MAPK inhibitor SB203580 resulted in a significant reduction of the IL-10-induced expression of GCR-α (Fig 5A), while the expression of IL-10R-α showed a non-significant trend to increase (Fig 5B).

**Fig 6:**
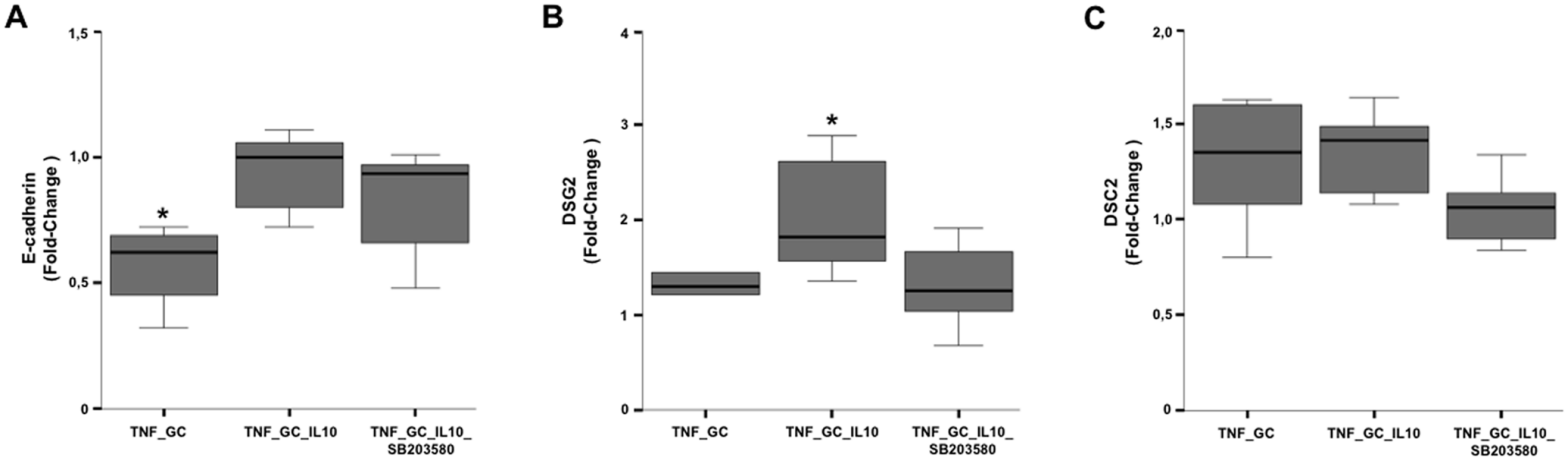
IL-10 plus GC increases DSG-2 expression dependent on p38 MAPK activity in Caco-2 cells. Graphs represent the fold change values (2^-AACt^) of E-cadherin (Panel A), DSG-2 (Panel B) and DSC-2 (Panel C) expression, with respect to their basal condition (control or DMSO group). *p≤ 0.05 *vs* other groups.

The expression of E-cadherin and DSG-2 significantly increased in the presence of IL-10, TNF-α and GC, with respect to monolayers with TNF-α and GC alone (Fig 6A and 6B). The addition of the inhibitor of p38 MAPK activity significantly reduced the expression of DSG-2 (Fig 6B), while it had no impact on that of E-cadherin (Fig 6A).

**Fig 7:**
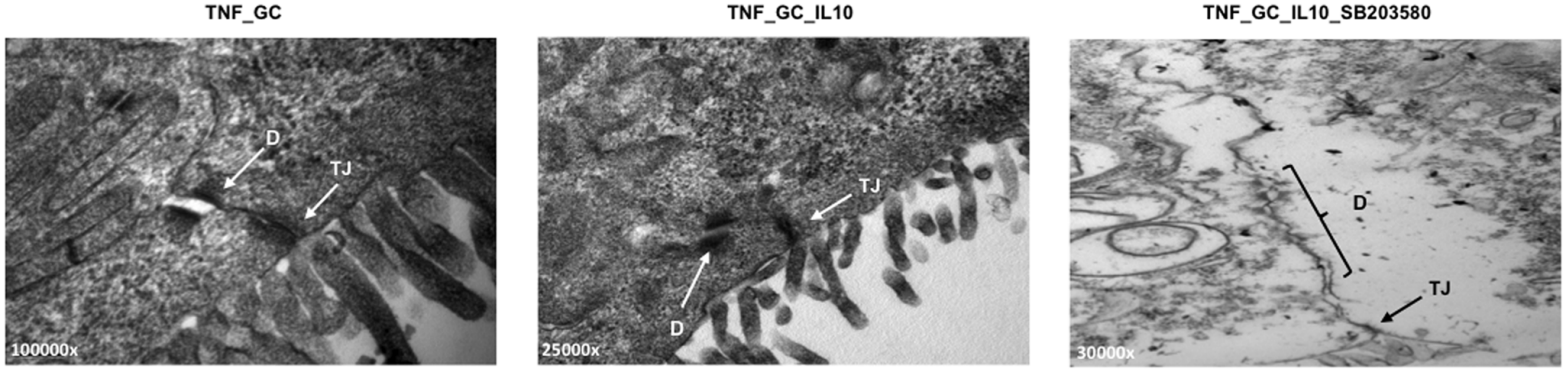
IL-10 and GC preserve p38 MAPK-dependent desmosome integrity and strength in Caco-2 monolayers. Representative transmission electron microscopy images of the paracellular junctions in Caco-2 cell monolayers treated with GC (Panel A), GC plus IL-10 (Panel B) or GC, IL-10 and SB203580 (Panel C) D: desmosomes; TJ: tight junctions. Magnification varies from 25,000 x to 100,000 x.

In order to ascertain if the results on the expression of paracellular junction-related molecules have a morphological correlation, we examined monolayers of confluent cells in the previous experimental conditions by transmission electron microscopy. We found that the TJ remained closed in all experimental conditions. However, the AJ and desmosomes (zones rich in cadherins) were usually open in TNF_GC group (57%), closed in the TNF_GC_IL-10 group (40%), and open again when the p38 MAPK activity was inhibited (60%) (Fig 7), but these differences were not statistically significant.

### Implications of the *in vitro* findings on GC responsiveness in UC

#### p38 MAPK phosphorylation is reduced in colonic epithelial cells from GC-refractory UC patients

**Fig 8:**
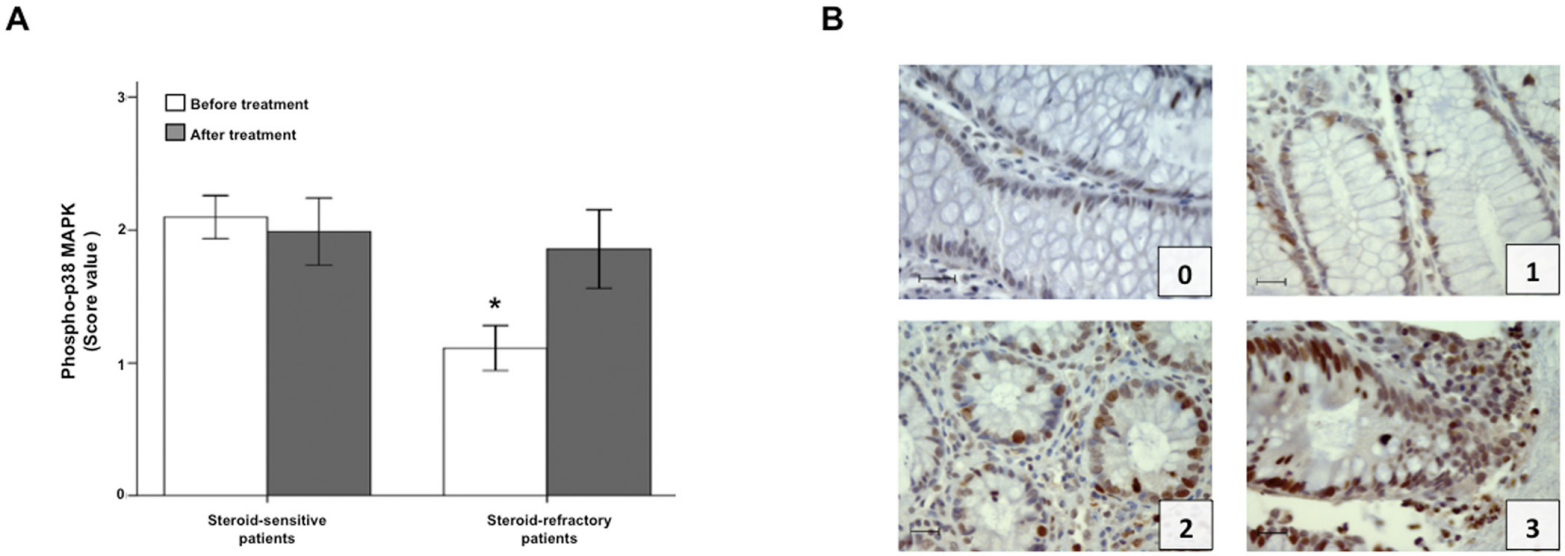
p38 MAPK phosphorylation is reduced in colonic epithelial cells from GC-refractory UC patients before treatment. Panel A shows the immunostaining score (mean±SEM) of phosphorylated p38 MAPK in colonic biopsies of steroid-responsive and steroid-refractory active UC patients, before and after GC treatment. *p = 0.028 *vs* steroid-sensitive patients before treatment. Panel B provides representative images from steroid-refractory UC biopsies (score value 0–1), and from steroid-sensitive UC biopsies (score value 2–3). Scale bar: 20 μm.

To understand the relationship of p38 MAPK with the response to GC in active UC, immunostaining of phosphorylated p38 MAPK was done in colonic biopsy samples from steroid-responsive (n = 8) and non-responsive (n = 7) UC patients, obtained before and after treatment with GC. Immunostaining for phosphorylated p38 MAPK in epithelial cells was predominantly nuclear. Despite the small number of patients, the number of positive nuclei in baseline biopsies was clearly decreased in GC-refractory patients (Fig 8A and 8B).

#### Changes of DSG-2 expression in the membrane of intestinal epithelial cells of patients with active UC

**Fig 9:**
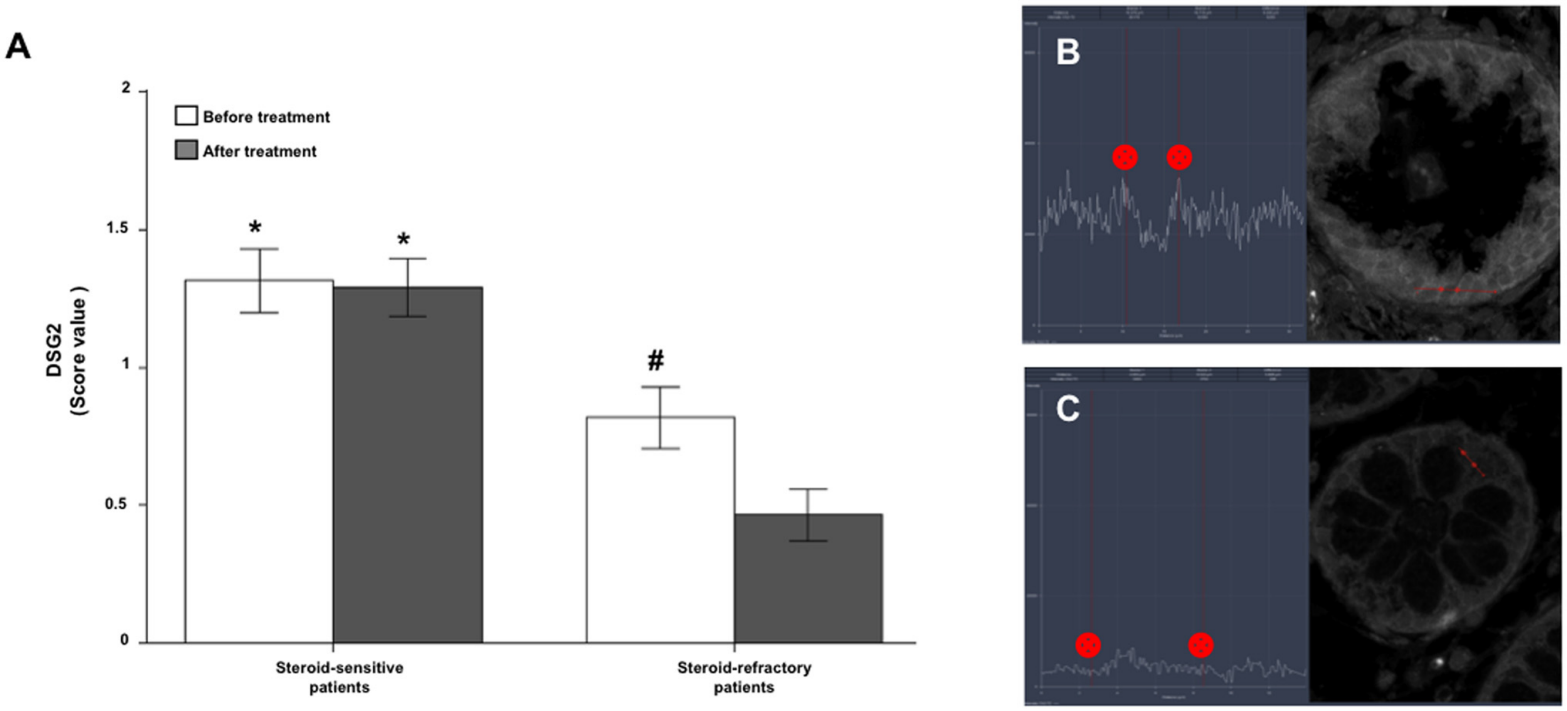
Steroid-sensitive UC patients show a greater DSG2 staining. Immunofluorescence staining score (mean±SEM) of DSG2 (Panel A) in colonic biopsies of steroid-sensitive and steroid-refractory UC patients, before and after treatment. Representative DSG2 staining intensity of steroid-sensitive (Panel B) and steroid-refractory (Panel C) patients are also provided. All images are 630 x. *p≤ 0.004 *vs* steroid-refractory patients, ^#^p = 0.023 *vs* after GC treatment.

Immunofluorescence for DSG-2 showed a greater intensity of staining in GC sensitive than in refractory patients, both before and after treatment (Fig 9). In addition, DSG-2 expression decreased with treatment only in GC refractory patients (Fig 9).

## Discussion

In this study, confluent Caco-2 cell cultures treated with TNF-α were used to investigate the effects of GC and IL-10 on the epithelial barrier function in inflammatory conditions. Caco-2 cell line undergoes in culture a process of spontaneous differentiation that leads to the formation of a monolayer of cells, expressing several morphological and functional characteristics of mature enterocyte, including functional paracellular junctions and well developed and organized microvilli on apical membrane [20]. These features makes it a good model of intestinal barrier.

The primary result of these experiments showed that the association of GC and IL-10 is able to revert the TNF-induced decrease of TEER (as a measure of epithelial integrity), an effect that does not occur when both agents are used separately (Fig 1). Such a synergistic action had not been previously described, despite that IL-10 therapy has been reported to improve intestinal epithelial function in a model of total parenteral nutrition [21], and GC alone prevent TNF-induced barrier disruption *in vitro* [22]. This result, together with our previous finding that steroid responsiveness is associated with the presence of IL-10 mRNA in the colon [3], argue for a necessary role of IL-10 on GC therapeutic effect on intestinal inflammation.

This role of IL-10 on epithelial barrier straightness in presence of GC appears to be associated to its ability to maintain some degree of p38 MAPK activation. In fact, IL-10 was able of maintaining some levels of activated p38 MAPK (Fig 2B), but TEER increased only when this cytokine was combined with GC, an effect that was abolished in the presence of an inhibitor of p38 MAPK activity (Fig 3). These findings suggest that some level p38 MAPK activation is necessary (but not sufficient) condition to maintain the integrity of the intestinal epithelial barrier. In this sense, IL-10 maintains a degree of phosphorylated-p38 MAPK that could favours the GC paracellular function.

Activated p38 MAPK has been traditionally associated to increased inflammatory activity in the gut of IBD patients [23,24]. However, experimental and clinical studies have shown conflicting results with respect to the role of p38 MAPK in the intestine. Clinical trials in active CD treated with inhibitors of p38 MAPK activity yield opposite results depending on the agent used [25,26]. Hollenbach *et al*. found that inhibition of p38 MAPK was able to drastically reduce colonic NF-kB activity with a consequent improvement of intestinal lesions in a murine model [27], whereas in other studies inhibition of p38 MAPK activation worsened the course of 2,4,6-trinitrobenzenesulfonic (TNBS) acid or dextran sulphate sodium (DSS) induced colitis [28,29].

These conflicting results could be explained by an opposite role of p38 MAPK in immune and epithelial cells. In fact, it has been reported that DSS- or TNBS-treated mice with myeloid cell-specific deletion of p38 MAPK alpha have less inflammation and an improved disease condition compared with wild-type mice, whereas mice with intestinal epithelial cell-specific deletion of p38 MAPK alpha had increased progression of colitis that resulted from disrupted intestinal epithelial homeostasis [30].

The protective action of p38 MAPK on the intestinal epithelium probably lies on its regulatory roles on apoptosis, and autophagy [31,32]. NOD2-dependent autophagy requires the activity of MEKK4 and p38 MAPK, independent of NFkB signalling [32]. RhoA/p38 MAPK axis contributes to mucosal healing through intestinal epithelial cell migration [33]. It has been shown that p38 MAPK activity mediated by probiotics in inflammatory conditions strengthens the TJ [34–36]. Moreover, it has been reported that IL-10-mediated p38 signalling inhibits inflammation-induced endoplasmic reticulum stress response mechanisms by modulating ATF-6 nuclear recruitment to the grp-78 gene promoter [13].

The IL-10/GC-mediated p38 MAPK effect on the cell-to-cell adhesion of the intestinal epithelial cells seems to depend on its translocation to the nucleus, as we found that the presence of both IL-10 and GC increased the number of nuclei positive for phosphorylated p38 MAPK in Caco-2 cells (Fig 4), a phenomenon not observed when the p38 MAPK activity inhibitor SB203580 was added. Moreover, the nuclear presence of phosphorylated p38 MAPK was also higher in intestinal pre-treatment biopsies of GC-responsive UC patients as compared to non-responsive ones (Fig 8). These observations, together with the finding of increased baseline IL-10 expression in the colon of UC patients who responded to GC [37], suggest that IL-10 induced nuclear translocation of active p38 MAPK could be a prerequisite for GC therapeutic response.

In an attempt to identify the biological action resulting from IL-10/GC-mediated p38 MAPK signalling, we investigated the expression of several possible target genes. Those of TJ proteins did not prove to be involved. However, the combination of IL-10 and GC was able to increase the expression of both E-cadherin and DSG-2 (a cadherin-like desmosomic protein) *in vitro*, but only the later was dependent on p38 MAPK activity. Since cadherins are the most abundant proteins in AJ and desmosomes, it is conceivable than TEER regulation via p38 MAPK would occur through these structures. Cadherins play a main role in regulating intestinal permeability by modulating the expression of Claudin-5 and regulating the function of Akt/FoxO [38].

Indeed, transmission electron microscopy analysis provided morphological confirmation of the involvement of desmosomes in paracellular epithelial sealing in presence of IL-10 and GC, an effect that did not occur when Caco-2 cells were treated with SB203580 (Fig 7). Desmosomes are key in the regulation of permeability. In fact, several studies demonstrate that blockade of DSG-2 or -3 by specific antibodies is capable of disrupting epithelial integrity and its barrier function [39–41].

GCRα expression ran in parallel to that of DSG-2, increasing in the presence of IL-10/GC, and returning to baseline when p38 MAPK phosphorylation was inhibited. This was not the case for IL-10Rα (and some proteins of its signalling pathway, such as SOCS-3 and IkBα, S1 Fig) which showed a further increase in their expression in the presence of SB203580.

Despite that the addition of IL-10 increases the expression of both GCRα and IL-10Rα in cellular monolayers, colonic UC biopsies showed lower amounts of both receptors in steroid-sensitive than in steroid-refractory patients. These apparently contradictory findings could be interpreted as an unsuccessful attempt of refractory patients to compensate for the lack of IL-10 (and hence impaired GC action) [37] by increasing the expression of both receptors.

In conclusion, this study demonstrates that the presence of IL-10 enhances the epithelial barrier via a mechanism that involves the phosphorylation of p38 MAPK, while favouring the expression and activity of IL-10 and GC alpha-receptors. The synergistic action of IL-10 and GC can favour recovery in injured intestinal epithelium of UC. This would probably occur by reducing the inflammation-induced intestinal epithelial cell stress, which helps to restore homeostasis and to isolate the *lamina propria* from luminal antigens. Furthermore, the molecular changes that affect the response to the GC can be detected in the intestinal epithelium of patients with UC. Anyway, the results obtained suggest that the intestinal epithelium is a potential target on the issue of inadequate response to GC in UC.

## Supporting Information

S1 Fig SOCS3 and IkBα p38 MAPK-independent expression in Caco-2 cell monolayers. Graphs represent fold change values (2^-AACt^) of SOCS3 (Panel A) and IkBα (Panel B) expression, with respect to their basal condition (control or DMSO group). *p≤ 0.05 *vs* TNF_GC_IL-10_SB203580 group.
(TIF)
