# Supplementary figures and images for: Interleukin-10 Enhances the Intestinal Epithelial Barrier in the Presence of Corticosteroids through p38 MAPK Activity in Caco-2 Monolayers: A Possible Mechanism for Steroid Responsiveness in Ulcerative Colitis
Source: PLoS One. 2015 Jun 19;10(6):e0130921. doi: 10.1371/journal.pone.0130921 (PMC4474693; doi:10.1371/journal.pone.0130921)

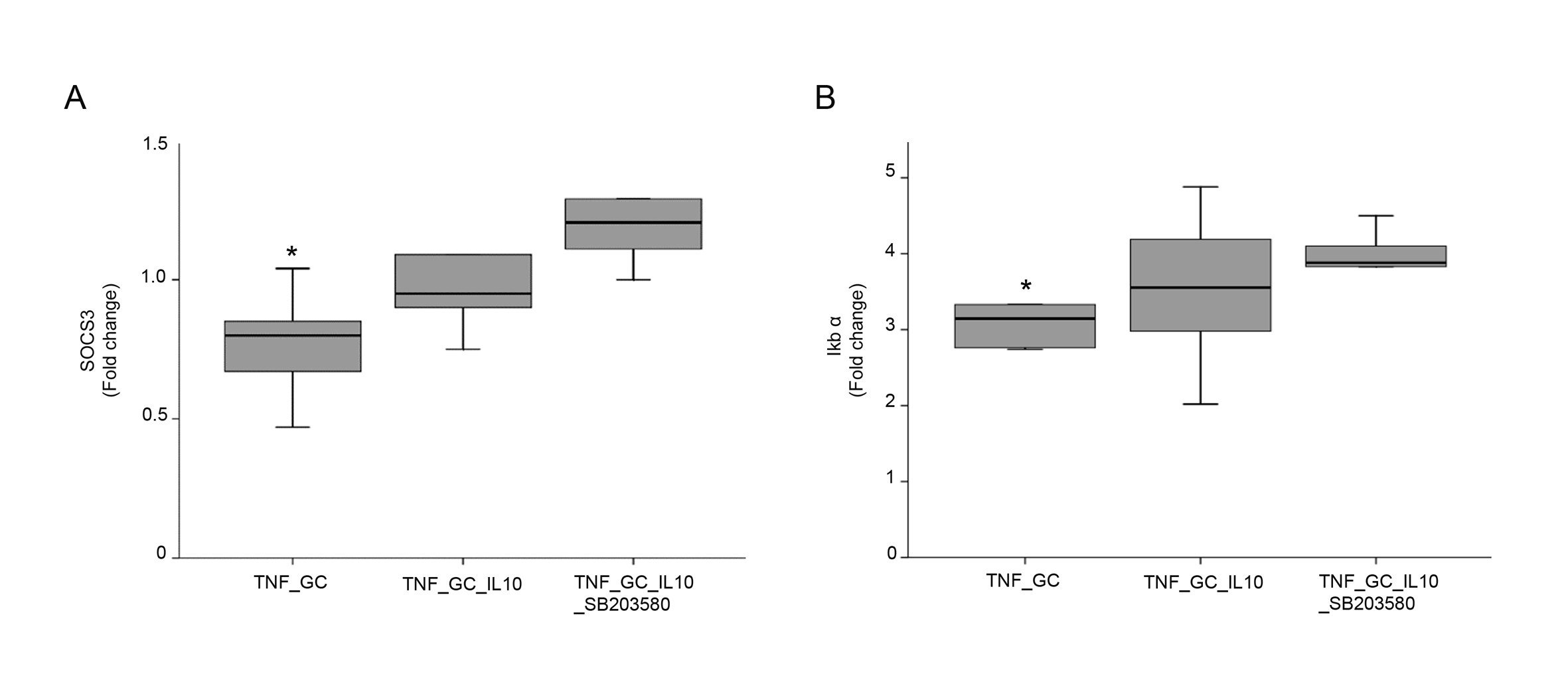

Supplement: S1 Fig — Graphs represent fold change values (2-AACt) of SOCS3 (Panel A) and IkBα (Panel B) expression, with respect to their basal condition (control or DMSO group). *p≤ 0.05 vs TNF_GC_IL-10_SB203580 group. (TIF) [file pone.0130921.s001.tif]
